# PLCε knockdown enhances the radiosensitivity of castration-resistant prostate cancer via the AR/PARP1/DNA-PKcs axis

**DOI:** 10.3892/or.2022.8433

**Published:** 2022-10-24

**Authors:** Jun Pu, Ting Li, Nanjing Liu, Chunli Luo, Zhen Quan, Luo Li, Xiaohou Wu

Oncol Rep 43: 1397–1412, 2020; DOI: 10.3892/or.2020.7520

Subsequently to the publication of this paper, an interested reader drew to the authors' attention that western blots featured in Figs. 4B and 5G (representing the ‘AR’ experiments in both cases) appeared to be the same, albeit that the bands were flipped vertically in Fig. 5G relative to Fig. 4B.

The authors have re-examined their data and realized that Fig. 5 was assembled incorrectly. The results from all the originally performed experiments were presented to the Editorial Office for our perusal. The revised version of Fig. 5, containing the correct data for the ‘AR’ experiment in Fig. 5G, is shown on the next page. The authors regret the inadvertent error that was made during the preparation of Fig. 5, and confirm that this error did not seriously affect the conclusions reported in the paper. The authors are grateful to the Editor of *Oncology Reports* for allowing them the opportunity to publish a Corrigendum, and all the authors agree to this Corrigendum. Furthermore, they apologise to the readership for any inconvenience caused.

## Figures and Tables

**Figure 5.**
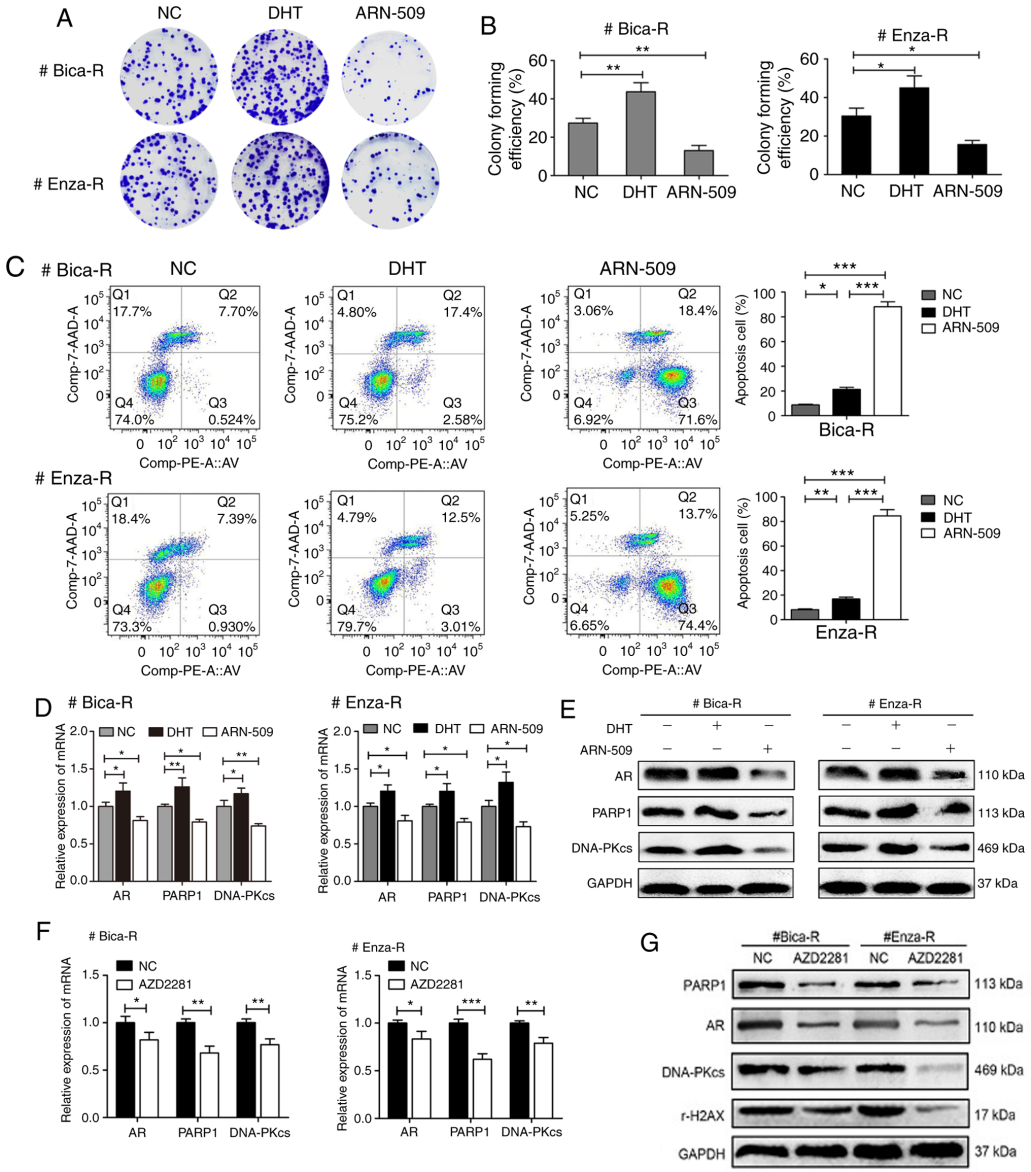
Role of an AR/PARP1 positive feedback loop in DNA damage repair in CRPC. PCa cells were treated with different inhibitors, and subsequently treated with 6 Gy of radiation. (A and B) Colony forming efficiency and (C) apoptosis analysis in treated PCa cells. (D and F) mRNA and (E and G) protein expression levels of AR, PARP1 and DNA-PKcs. *P<0.05, **P<0.01 and ***P<0.001. PCa, prostate cancer; AR, androgen receptor; PARP1, Poly (ADP-ribose) polymerase 1; CRPC, castration-resistant PCa.

